# Autophagy Contributes to the Death/Survival Balance in Cancer PhotoDynamic Therapy

**DOI:** 10.3390/cells1030464

**Published:** 2012-08-03

**Authors:** Valentina Inguscio, Elisa Panzarini, Luciana Dini

**Affiliations:** Department of Biological and Environmental Science and Technology (Di.S.Te.B.A.), University of Salento, Lecce 73100, Italy; Email: valenting@hotmail.it (V.I.); elisa.panzarini@unisalento.it (E.P.)

**Keywords:** photodynamic therapy (PDT), autophagy, cancer therapy, Atg proteins, immunogenic cell death, cell death

## Abstract

Autophagy is an important cellular program with a “double face” role, since it promotes either cell survival or cell death, also in cancer therapies. Its survival role occurs by recycling cell components during starvation or removing stressed organelles; when damage becomes extensive, autophagy provides another programmed cell death pathway, known as Autophagic Cell Death (ACD). The induction of autophagy is a common outcome in PhotoDynamic Therapy (PDT), a two-step process involving the irradiation of photosensitizer (PS)-loaded cancer cells. Upon tissue oxygen interaction, PS provokes immediate and direct Reactive Oxygen Species (ROS)-induced damage to Endoplasmic Reticulum (ER), mitochondria, plasma membrane, and/or lysosomes. The main biological effects carried out in cancer PDT are direct cytotoxicity to tumor cells, vasculature damage and induction of inflammatory reactions stimulating immunological responses. The question about the role of autophagy in PDT and its putative immunological impact is hotly controversial and largely studied in recent times. This review deals with the induction of autophagy in PDT protocols and its dual role, also considering its interrelationship with apoptosis, the preferential cell death program triggered in the photodynamic process.

## 1. Introduction

Exposure of tumor cells to PhotoDynamic Therapy (PDT) leads to cell death through multiple pathways. This ability makes PDT a promising cancer therapy, as chemotherapy and ionizing radiation, and many studies have been performed in this context, recently summarized by Kessel and Oleinick [1]. 

Among the therapeutic modalities used in cancer clinical trials, PDT is a Reactive Oxygen Species (ROS)-mediated process, resulting in the rapid initiation of cell death only in malignant cells without affecting the healthy cells surrounding the tumor area. ROS, generated upon visible light exposure of cancer cells pre-loaded with a photosensitizing agent (PS), trigger a photodynamic stress that concomitantly activates multiple signaling cascades depending on the ROS subcellular location and intensity of the oxidative damage. The cells will “decide” to survive or to die. In this context, the role played by autophagy and its interrelationship with apoptosis is very crucial. In fact, due to its enigmatic role in cytotoxicity or cytoprotection in cells, also in PDT, autophagy is a factor mediating two response endpoints: photodamaged cellular components repair and cell demise. 

This report will review data about the involvement of autophagy in PDT outcomes, mainly focusing on its pro-survival or pro-death role and the crosstalk with apoptosis. 

## 2. Autophagy: A Triple-Faced Coin

Autophagy is a complex ‘self-eating’ mechanism that, depending on the specific circumstances, constitutes a cell survival strategy to stress conditions avoiding cell death or an alternative ‘self-killing’ pathway, referred to as Autophagic Cell Death (ACD) or type II Programmed Cell Death (PCD), initiated by persistent catabolic reactions ending in the metabolic and bioenergetic cell collapse. 

The autophagic process involves the sequestration of damaged organelles and long-lived misfolded/ggregated proteins within double-membrane vesicles, termed autophagosomes, delivered to the cell’s own lysosomal degradation. The autophagosomes engulfing intracellular contents fuse with lysosomes to form autophagolysosomes, where lysosomal hydrolases degrade the sequestered material recycling it.

In mammals, three different types of autophagy have been identified: macroautophagy, microautophagy and chaperone-mediated autophagy (CMA) [2]. Macroautophagy (hereafter referred to as autophagy) is a non-specific bulk degradation process which implies the formation of a double-membrane around a targeted intracellular portion, separating the enclosed components from the rest of the cytoplasm. The resultant vesicle then fuses with a lysosome, whose hydrolases subsequently degrade the contents.

Microautophagy is a nonselective form of autophagy involving the direct engulfment of cytoplasmic cargo at the lysosomal surface, by invagination, protusion, and/or septation of the lysosomal/vacuolar membrane. Its exact function has not been elucidated, although it is thought to contribute to the maintenance of lysosomal limiting membrane size [3].

Chaperone-mediated autophagy (CMA) is the only selective form of autophagy modulating the turnover of specific cytosolic proteins containing a pentapeptide motif. Unlike the other two forms of autophagy, CMA implies the selective recognition of substrates by cytosolic chaperones and their translocation across the lysosome membrane on a one-by-one basis in a receptor-mediated manner [4]. Particularly, the pentapeptide motif is recognized by the chaperone Heat shock cognate 70 (Hsc70), which delivers the protein substrates to the surface of lysosomal membrane where, through interaction with the receptor lysosome-associated membrane protein 2A (LAMP-2A), they are translocated into the lysosomal lumen for rapid degradation.

The three main autophagic pathways function as part of a global program for the degradation of cell's own components by the lysosomal machinery.

## 3. Autophagic Molecular Core Machinery

**Figure 1:**
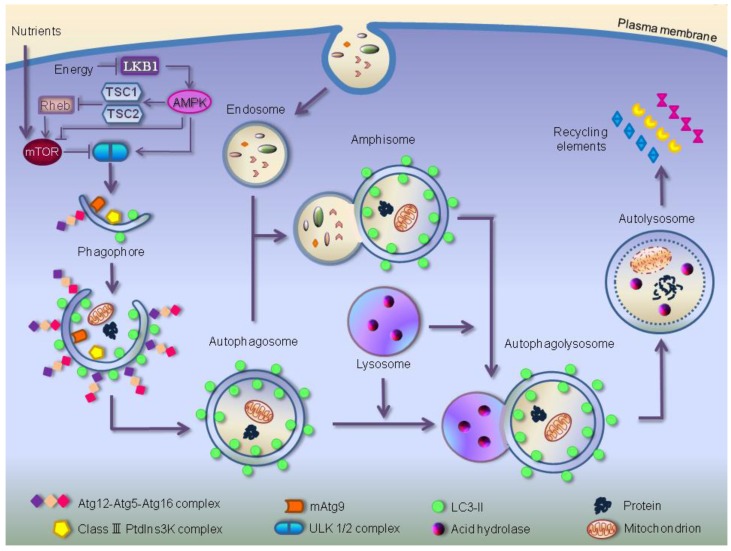
The process of autophagy in mammalian cells. Autophagy is initiated by the formation of the phagophore, mediated by the class-III phosphatidylinositol 3-kinase (PtdIns3K) complex that includes hVps34, the yeast Vps15 homologue, p150, Beclin1, and human Atg14/Barkor, and followed by a series of steps. These include the elongation and expansion of the phagophore, closure of a double-membrane autophagosome engulfing material targeted for degradation, such as damaged organelles and long-lived misfolded/aggregated proteins, autophagosome maturation through fusion with an endosome (the product of fusion is known as an amphisome) and/or lysosome (the product of fusion is known as an autophagolysosome), degradation of the vesicle content by acid hydrolases inside the autolysosome and recycling of the degradation products (amino acids, lipids and sugars) for ATP production. Autophagy induction is mediated by the kinase activity of ULK1/2 complex. Energy depletion causes the phosphorylation and activation of AMP-activated protein kinase (AMPK) by the serine/threonine kinase LKB1. AMPK directly phosphorylates the tuberous sclerosis complex 1/2 (TSC1-TSC2), leading to the inactivation of the small GTPase Rheb, which in unstressed cells binds and activates the master regulator of autophagy, mTOR (mammalian target of rapamycin kinase), inducing autophagy. AMPK also associates with ULK1, playing an important role in autophagy induction. The core molecular machinery also requires the membrane carrier mAtg9, delivering membrane lipids to the forming autophagosome, and two conjugation systems for the elongation and extension of the phagophore membrane: LC3-II, which is formed as a result of the Atg4-mediated cleavage of cytosolic LC3, and the Atg12–Atg5–Atg16L complex, mediating LC3-II recruitment to the membrane.

The autophagic process is initiated by the formation of an isolation membrane, sometimes referred to as phagophore in mammals, which curves around part of the cytoplasm to form a closed double membrane vesicle, the autophagosome (Figure 1). 

The *Atg* (AuTophaGy-related) family genes regulate the rearrangement of subcellular membranes enwrapping intracellular material for lysosomal degradation. This subset of numerically designated genes was first characterized in the yeast *Saccharomyces cerevisiae*, but mammalian orthologues have been successively identified. 31 Atg proteins orchestrate the molecular autophagic process in yeast and most of them (Atg1-10, Atg12-14, Atg16-18, Atg29, and Atg31) are required for the nucleation and elongation of the isolation membrane and the autophagosome formation [5]. The Atg proteins can be classified into different complexes that collectively make up the autophagic core machinery, including two kinase complexes, two ubiquitin-like conjugation systems and a shuttling protein. 

The first complex consists of the serine/threonine protein kinase Atg1, which interacts with the autophagy regulatory proteins Atg13 and Atg17, and it is negatively regulated, in yeast, by the protein kinase target of rapamycin (TOR). In mammals, the two Atg1 orthologues, ULK1 and ULK2 (Unc51-Like Kinase), along with the putative mammalian counterparts of Atg13 (mAtg13) and Atg17 (focal adhesion kinase family–interacting protein of 200 kDa, FIP200) are under the tight regulation of the mammalian target of rapamycin (mTOR) [6]. 

The second multimolecular kinase complex, including the class-III phosphatidylinositol 3-kinase (PtdIns3K), Vps34 (Vesicular protein sorting 34), its regulatory subunit Vps15, Atg6 and Atg14 [7], is required for autophagy induction and initiation of autophagosome formation [8]. It mediates the correct localization of other autophagic proteins to the vacuolar membrane referred to as the Pre-Autophagosomal Structure or Phagophore Assembly Site (PAS), the yeast perivacuolar site where the autophagy machinery is concentrated [9]. In mammalian cells, hVps34, the Vps15 homologue, p150, the Atg6 orthologue, Beclin1, and human Atg14/Barkor are also essential for autophagy [10]. Autophagosome formation requires the activity of the PtdIns3K/Vps34, since it is involved in the mechanism for increasing the size of the sequestering membrane. Indeed, the chemical inhibition of this activity by 3-methyladenine (3-MA) or wortmannin prevents the composition of new autophagosomes [11].

Two ubiquitin-like conjugation systems are important for autophagosome elongation. The first one involves Atg5, Atg12 and Atg16 proteins and the other requires the conjugation of microtubule-associated protein 1 light chain 3 (MAP1-LC3, also known as LC3), a mammalian homolog of yeast Atg8, to the lipid phosphatidylethanolamine (PE). The covalent attachment of Atg12 to Atg5 is mediated by Atg7 (an ubiquitin-activating E1-like enzyme) and Atg10 (an ubiquitin-conjugating E2-like enzyme) [12]. The Atg12-Atg5 conjugate further interacts with Atg16 to form a new complex transiently localizing to the elongation membrane, until autophagosome completion [13]. The mature autophagosome excludes Atg12-Atg5-Atg16 complex, which in turn is responsible for the recruitment to the membrane of LC3-II, the product of the second ubiquitin-like conjugation [14]. The cytosolic precursor protein LC3 is cleaved by the cysteine protease Atg4 to generate cytosolic LC3-I, which is conjugated to PE to form membrane-associated LC3-II (or LC3-PE). The LC3-I lipidation is catalyzed by the E1-like enzyme Atg7 and E2-like enzyme Atg3, while Atg5-Atg12–Atg16 complex acts as an E3-like ligase specifying the site of LC3 lipidation [13,14]. After fusion of the autophagosome with the lysosome, a second cleavage by Atg4 delipidates LC3-II, reverting it back into cytosolic LC3-I, which dissociates from the outer autophagosomal membrane [15]. LC3-II is widely used to monitor autophagy due to its specific association with autophagosomes, whose intracellular number correlates with LC3-II levels [16].

The shuttling protein Atg9 is the only integral transmembrane protein required for autophagosome formation. In *S. cerevisiae*, Atg9 cycles between peripheral sites and the PAS, suggesting that it may function as membrane carrier in autophagy. It is considered the best candidate marker of the autophagosome membrane source, which includes Endoplasmic Reticulum (ER), Golgi complex and mitochondria as lipid ‘donator’ organelles for autophagosomes [17,18]. mAtg9, the mammalian homologue of yeast Atg9, has been proposed to deliver lipids to the forming autophagosome cycling between the trans-Golgi-Network (TGN) and endosomes in an ULK1-dependent manner [19].

Prior to its fusion with lysosome, the complete autophagosome undergoes a stepwise maturation regulated, in mammalian cells, by the small GTPase Rab7, a central mediator of vesicular trafficking [20]. The autophagosome starts its maturation by fusion with vesicles originating from early endosomes, to form a structure referred to as amphisome; next it fuses with protease-containing late endosomal compartments. The final maturation step is the fusion of autophagosome with lysosome to form the ‘autolysosome’, a process assisted by lysosomal receptor proteins LAMP1 and 2. LAMP2 deficiency results in the accumulation of autophagosomes, due to a defect in the last fusion step [21]. Within the lysosome, a cocktail of hydrolytic enzymes, including cathepsins B, D and L [9], promotes the degradation of the sequestered material, completing the catabolic process [22].

Similarly to yeast, the classical signaling pathway that regulates autophagy in mammalian cells involves the serine/threonine kinase, mammalian target of rapamycin, mTOR, a negative control of autophagy [23]. mTOR activity is inhibited by nutrient starvation, a critical step for autophagy induction in eukaryotes. In the presence of nutrients, mTOR is associated with the large complex containing the mammalian homolog of an Atg gene product (mAtg13), ULK1, ULK2 and the scaffold protein FIP200. Starvation conditions lead to mTOR dissociation from this complex resulting in the partial dephosphorylation of mAtg13 and ULK1/2 and in the FIP200 phosphorylation [6]. This constitutes a strong signal for the induction of autophagy, which promotes cell survival until nutrients become available again.

It has been reported that, in mammals, metabolic and energy stresses elicit autophagy through activation of AMP-activated protein kinase (AMPK), energy sensor in ATP and/or glucose starvation [24]. 

AMPK induces autophagy by inhibiting Target-Of-Rapamycin Complex-1 (TORC1), a multiprotein complex sensitive to rapamycin, containing mTOR, GβL, PRAS40 and Raptor [25], by phosphorylating Tuberous Sclerosis Complex 2 (TSC2) tumor suppressor [26] and/or Raptor [27]. Recently, it has been suggested that activated AMPK can trigger the autophagy by association with ULK1-mTORC1 complex [28] and phosforylation of Raptor [29]. Alternatively, AMPK can directly phosphorylate ULK1 bypassing mTOR inhibition [30,31]. However, different sites of phosphorylation have been identified: S467, S555, T574, S637 [30] and S467, S317, S637, S777 [31]. Moreover, Loffler and coworkers demonstrate that ULK1 can, in turn, block AMPK by phosphorylation, representing a negative feedback circuit in autophagy induction [32].

In mammals, the autophagic process undergoes a regulation by Beclin1, a protein able to promote autophagy and inhibit tumorigenesis [33]. The detailed molecular mechanism involving Beclin1 in autophagy is yet enigmatic, in particular its involvement in autophagosome formation and membrane trafficking is not fully understood [34,35]. Beclin1 and its interacting partner, class III PtdIns3K/hVps34, cooperate in the generation of phosphatidylinositol 3-phosphate, a lipid second messenger essential for autophagosome formation [36].

Beclin 1 contains three functional domains: a short BH3 motif, able to bind Bcl-2 family proteins (as below reported), a coiled-coil segment, and a terminal domain so-called Evolutionarily Conserved Domain (ECD), potentially involved in initiating the autophagosome formation. In fact, Huang *et al*. [37] recently suggest that Beclin1 ECD associates with lipid membrane through its three consecutive aromatic fingers ensuring the curvature of membranes involved in autophagosome formation mechanism, such as mitochondria [18] and ER membranes [38]. 

It is well known that intracellular Ca^2+^ regulates autophagic process. However, the Ca^2+^ role is yet ambiguous [39], since it can be inhibitory [40,41,42,43,44,45] or stimulatory [46,47,48,49,50,51]. IP3 and its receptors (IP3Rs) mediate both Ca^2+^inhibition and stimulation effect. Recently, Decuypere *et al.* [52] demonstrate that IP3R-mediated Ca^2+^ signaling and autophagy induction processes are interdependent. In fact, starvation induces an early transient sensitization of both the machinery Ca^2+^ signaling and IP3R suggesting that autophagy stimulation depends on proper IP3R-mediated Ca^2+^ signaling since, blocking IP3R, LC3 lipidation is abolished. In healthy cells, Ca^2+^ released *via* IP3Rs localized on ER membrane is taken up by mitochondria stimulating mitochondrial ATP production, which in turn inhibits autophagy through AMPK [53]. Moreover, Vicencio and coworkers [42] suggest the existence of a complex including IP3R, Beclin1 and Bcl2. Particularly, IP3Rs could decrease the amount of free Beclin1 by anti-autophagic complexes Beclin1-Bcl2 formation.

In stressed cells, ER Ca^2+^ signaling is enhanced with consequent [Ca^2+^]_cyt_ increase. The [Ca^2+^]_cyt_ activates calmodulin that in turn triggers two autophagic pathways, *i.e.*, AMPK-dependent inhibition of mTOR [48,51,54] and dissociation of Beclin1 from Bcl2 *via* DAPK-mediated phosphorylation of Beclin1 [55,56].

Particularly it has been hypothesized that IP3R operates as a scaffold binding Beclin1 and Bcl-2 separately. In non-starved cells, Beclin1 is kept at the ER membrane in the proximity of the IP3R by Bcl-2 interaction or by binding to the suppressor domain on IP3R. Conversely, in starved cells, Beclin1 shuttles from Bcl-2 to IP3R directly binding them and sensitizing Ca^2+^ signaling, that in turn leads to exit of Ca^2+^ in cytosol [52]. 

Ca^2+^ is also involved in autophagolysosome formation. Indeed, upon autophagy induction the TRPML3 Ca^2+^-permeable channel is overexpressed on autophagosomes membrane and acts on membrane trafficking involved in autophagic flux by regulating the fusion of autophagosomes with lysosomes [57]. 

## 4. Autophagy: From Survival Program to Cell Death Mechanism

### 4.1. Autophagy between Life and Death

Autophagy is an evolutionarily conserved catabolic pathway primarily functioning as cell survival adaptive mechanism in different conditions, such as protein aggregate-induced stress, starvation, ER stress and pathogen infection. At its basal levels, autophagy ensures the cellular energy homeostasis through the disposal of damaged organelles and proteins.

The pro-survival function of autophagy represents an ancient stress tolerance mechanism conserved from yeast to mammals. In the mammalian system, the loss of function of genes essential for autophagy results in a death signal. Particularly, mice deficient of Beclin1 die during early embryonic development [36], while mice lacking Atg5 die within one day after the birth [58]. Moreover, mice with Atg7 deficiency develop neurodegenerative disorders involving ubiquitin-containing inclusion bodies [59].

Other autophagic programs promoting cell survival involve the removal of damaged organelles and degradation of intracellular pathogens and protein aggregates [60,61]. During nutrient and growth factor deprivation, the increased levels of autophagy protect cells from apoptosis allowing cell survival [62,63]. Cells can employ the autophagic process to survive Mitochondrial Outer Membrane Permeabilization (MOMP) and the release of mitochondrial apoptogenic proteins, including cytochrome c [64].

Paradoxically, in certain conditions, autophagy can also kill cells. Indeed, massive or unregulated autophagy may induce cell death due to an excessive self-degradation of essential cytoplasmatic substrates acting as an alternative form of PCD: Autophagic Cell Death (ACD) or type II PCD [65]. ACD is associated with the occurrence of increased numbers of autophagosomes, giving the cell a characteristic vacuolated appearance [66]. This PCD form differs from other cell death mechanisms, such as apoptosis and necrosis. Unlike apoptosis whose activation is mediated by the cleavage of caspases, the executioners of the apoptotic program, ACD is usually thought as a caspase-independent pathway. On the other hand, ACD involves the activation of lysosomal cathepsins, the main ‘self- destroyer’ mediating cell integrity compromise typically occurring during necrosis [67].

Autophagy can emerge as cell death mechanism in cells with genetic defects in the apoptosis machinery and signaling [68] or in the presence of caspases inhibitors [69]. This suggests that, in some settings, autophagy can act as a molecular backup mechanism to execute cell death when apoptosis is inhibited. Conversely, accumulating evidence reveals that the inhibition of autophagy promotes apoptotic cell death [70]. Moreover, in apoptosis-deficient cells, the inhibition of autophagy sensitizes cells to necrotic cell death program [68]. 

Autophagy fails to preserve cell viability also in response to massive oxidative stress mediated by elevated levels of ROS, the highly reactive molecules deriving from the incomplete reduction of oxygen [71]. Conditions of starvation increase levels of ROS that modulate the autophagic process as a survival pathway. However, the massive ROS production upon treatment, for example, with various anti-cancer drugs switches the autophagic defense response against oxidative stress into a lethal *stimulus* leading to ACD. The ponderous ROS accumulation has been reported to depend on the selective autophagic degradation of the enzymes catalyzing the decomposition of ROS, named catalases [72].

Moreover, the default pro-survival role of autophagy in cultured cells exposed, for example, to ER stress, caspase inhibition, oxidative stress, growth factor deprivation, interferon-γ, anti-cancer drugs, p53 activation, oncogene activation and radiation [68,69,72,73,74,75,76,77,78,79] is inhibited by depletion of *Atg* genes, the key regulators of autophagy core machinery, ending in cell death increase.

### 4.2. Cross-Talk between Autophagy and Apoptosis

Although ACD occurs in non-physiological conditions, such as chemical inhibition, massive autophagic flux or apoptosis deficiency, and it remains still unclear if it happens in physiological circumstances, apoptosis and ACD may be co-regulated. Indeed, several evidences suggest mutual interconnections between these two forms of PCD. 

At the molecular level, the cross-talk between apoptosis and autophagy is orchestrated by numerous genes affecting both pathways. These include genes such as p53 as well as some of the basic machinery that regulate the death programs (e.g., Atg5, Bcl-2).

The tumor suppressor p53, a known activator of apoptosis, can also induce autophagy through increased expression of DRAM (Damage-Regulated Autophagy Modulator), a p53 target gene encoding a lysosomal protein that induces macroautophagy, as an effector of p53-mediated death [80]. Depending on its localization, p53 promotes the induction of autophagy, leading to cell death, when it is located in the nucleus; conversely, cytoplasmic accumulation of p53 represses basal pro-survival autophagy.

Atg5, a critical regulator of autophagosome precursor synthesis, can also influence apoptotic signaling pathways. Upon an apoptotic *stimulus*, Atg5 is cleaved by calpain to form a N-terminal cleavage product that translocates to the mitochondria. The truncated form of Atg5 may inactivate the anti-apoptotic activity of Bcl-x_L_ by displacing Bcl-x_L_-Bax complexes, thereby promoting Bax oligomerization. The resulting caspase activation blocks Beclin1-dependent autophagosome synthesis by cleaving Beclin1 [81]. This cross-talk could represent a mechanism by which apoptosis suppresses autophagy.

Bcl-2 family proteins, well known apoptosis regulators including pro- and anti-apoptotic members, also control non-apoptotic programmed cell death that depends on the autophagic genes. Particularly, the anti-apoptotic Bcl-2 protein inhibits starvation-induced autophagy by its direct interaction with Beclin1 [82]. In fact, Beclin1, the mammalian orthologue of yeast Atg6, possess a Bcl-2 homology-3 (BH3) binding domain [83] allowing its interaction with the anti-apoptotic proteins of the Bcl-2 family, such as Bcl-2 and Bcl-x_L_. Although Beclin1 is able to bind Bcl-2, it cannot counteract its anti-apoptotic function at the mitochondrial membranes level. Conversely, Bcl-2 or Bcl-x_L_ reduces the pro-autophagy activity of Beclin1. Interestingly, only ER-targeted Bcl-2 (or Bcl-x_L_), but not mitochondria-targeted Bcl-2 (or Bcl-x_L_), inhibits starvation-induced autophagy [83]. The constitutive Bcl-2/Bcl-x_L_–Beclin1 association is disrupted by signals promoting autophagy. DAPk (Death-Associated Protein kinase)-mediated phosphorylation in the BH3 domain of Beclin1 promotes dissociation of Beclin1 from Bcl-x_L_ to induce autophagy [56]. Similarly JNK (c-jun N-terminal kinase)-mediated phosphorylation of Bcl-2 results in its release from Beclin1 regulating starvation-induced autophagy [84].

## 5. Autophagy and Cancer

The balance and complex interplay between apoptosis and autophagy are not only intriguing intellectual curiosities for molecular researchers, but they also represent an exploitable topic in cancer research [85].

Autophagy acts as a double-edged sword in the setting of cancer [86]. Indeed, depending on the different stages of cancer progression, it can function as a tumor suppressor (at the early stage of tumor development) or promote tumor progression (at the advanced stages of tumorigenesis) [87]. 

The assumption that autophagy exerts a tumor suppressor mechanism relies on the finding that it is frequently downregulated in tumors [88]. This is probably due to the upregulation of class I phosphatidylinositol 3-kinase pathway in cancer cells. Beclin1 has been reported as a plausible tumor suppressor, since allelic Beclin1 deletion is frequently observed in human breast, ovarian and prostate cancer. Moreover, Beclin1^+/−^ mice were shown to be tumor prone, suggesting that Beclin1 is a haploinsufficient tumor suppressor gene [89]. Counterintuitive to its tumor suppressive role, autophagy can act as a protector of cancer cell survival [86,90]. In fact, because of its ability to supply nutrients from breakdown cytoplasm, autophagy enables tumor cells to tolerate stress conditions, including hypoxic microenvironment, starvation and probably some cancer treatment regimens. Autophagy upregulation upon cellular detachment from extracellular matrix sustains cell viability also in metastasizing cells [91]. Knockdown of essential autophagy genes in tumor cells has been shown to confer or potentiate the induction of cell death [86].

Understanding the role of autophagy in cancer treatment is pivotal since many cancer therapies using cytotoxic drugs or other anti-neoplastic and pharmacological agents have been shown to induce autophagy in tumor cells. Particularly, features of ACD, underlined by the accumulation of autophagosomes, were observed in cancer dying cells in response to cancer treatment, such as conventional therapies and recent cancer regimens exploiting selective photosensitizing agents [92]. 

## 6. Photodynamic Therapy Fundamental Aspects

**Figure 2:**
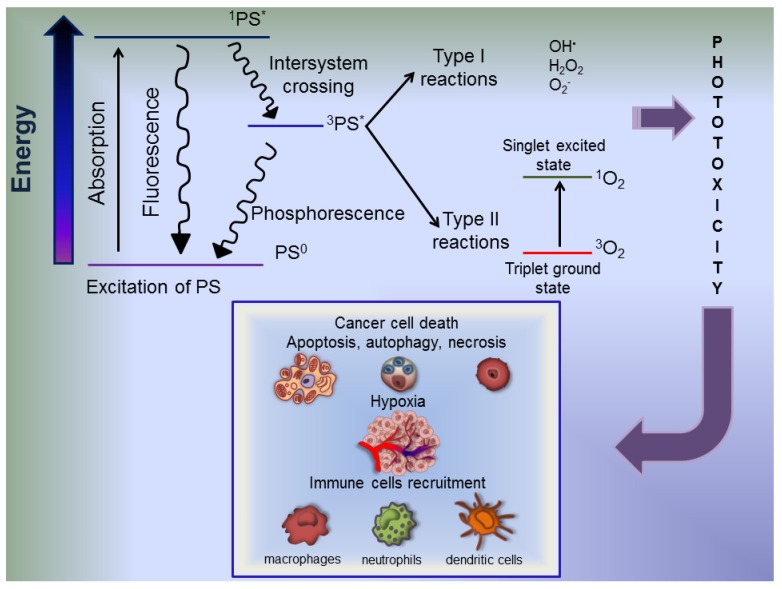
Photophysical and photochemical mechanisms in PhotoDynamic Therapy (PDT) and their effects on tumor. The basis of PDT is the toxic photodynamic reactions induction leading to ROS generation by photochemical and photophysical processes. In the dark, photosensitizers (PSs) exist in the ground state (PS^0^) with paired electrons; upon absorption of a light photon, one of these electrons shifts to a higher energy orbital, inverts its spin and forms the extremely unstable singlet excited state, ^1^PS^*^, with a half-life ranging between 10^−6^ and 10^−9^ seconds. The ^1^PS^*^ can decay back to the ground state, emitting fluorescence, or undergo intersystem crossing to the longer lived (10^−3^ second) triplet excited state (^3^PS^*^), where two electrons are unpaired and have the same spin. ^3^PS^*^ may, in turn, decay back to the ground state, emitting phosphorescence or trigger two competing types of photochemical reactions inducing phototoxicity. In type-I photochemical reaction, the ^3^PS^*^ reacts directly with cell components transferring an electron or hydrogen to water or biomolecules to form peroxides, superoxide ions and hydroxyl radicals. In type-II photochemical reaction, the ^3^PS^*^ mediates the energy transfer process with molecular oxygen (^3^O_2_) generating a highly toxic activated oxygen molecule, the singlet oxygen (^1^O_2_). In PDT, the *in situ* generation of ^1^O_2_ plays a key role in photodynamic cytotoxicity because of its efficient interaction with a large variety of biomolecules, such as proteins, nucleic acids and lipids. The cancerous tissue/cells obliteration is ensured by the destructive power of ROS generated upon visible light irradiation of the PS. This result is gained by a multifactorial impact, including direct and indirect effects. In fact, phototoxicity on neoplastic cells occurs via direct tumor cell death by apoptosis and/or autophagy and/or necrosis, damage to the vasculature leading to tumoral area hypoxia and rapid recruitment and activation of immune cells, such as macrophages, neutrophils and dendritic cells. These cells release cytokines amplifying the immune response by activation of B and T cells recruited to the site of tumor.

Cancer PhotoDynamic Therapy (PDT) is a novel therapeutic modality based on the oxidative damage to cellular components following ROS generation upon visible light activation of a cell-localized PhotoSensitizer (PS). The interaction between light, cell or tissue molecular oxygen and PS gets the photodynamic reaction [93]. The PS subcellular localization dictates the primary site of damage and the consequent outcome of the treatment, implying direct cell damage and secondary effects. The first induces cytotoxicity triggering apoptotic and/or necrotic and/or autophagic cell death in different cell types [94]. Conversely, secondary effects involve damage to the tumor vasculature and inflammatory reaction induction leading to the development of systemic immunity [95] (Figure 2). 

PSs are light-absorbing substances able to initiate photochemical or photophysical reactions in another molecule. PSs are not consumed in these reactions. PDT efficacy mainly depends on PS physico-chemical properties and pharmacokinetic/toxicity features, such as chemical purity, selectivity for cancer cells, chemical and physical stability, activation at wavelengths ensuring optimal tissue penetration, short time interval between administration and maximal accumulation within tumor tissues and rapid excretion to minimize photosensitivity period [96]. For the PS administration very important is its solubility; in fact, amphiphilic PSs are very easy delivered in the blood, they do not form precipitated and/or aggregated, and they effectively penetrate through cell membrane phospholipid bilayer. In cancer therapy, PS is capable to accumulate in tumor but not in healthy cells, thus ensuring photo-induced toxicity only in the target pathological tissue [97]. In fact, tumor area has several characteristics leading to accumulation of PSs, such as low pH of the neoplastic extracellular compartment, increased low-density lipoprotein receptor expression in proliferating endothelial/cancer cells and reduced lymphatic drainage in neoplasia [98]. An ideal PS should not be toxic *per se* and should not generate toxic degradation products upon irradiation. PSs can be generally classified on the basis of generation, synthetic purity, targeting and chemical structure [97].

In PDT can be used several light sources, *i.e.*, lasers, lamps and Light Emitting Diodes (LED), whose main characteristic should be a spectrum coincident with the maximum absorption wavelength range of the PS chosen. Particularly, the lasers ensure both the exact selection of wavelengths and the precise application of light, while LEDs are versatile in light delivery on difficult anatomic area [99].

Upon irradiation, the PSs initiate a photodynamic reaction based on photophysical and photochemical processes. In particular, the PS in the ground state absorbs light and is activated to the single excited state with a short half-life ranging from 10^−6^ to 10^−9^ seconds. The singlet excited PS either decays back to the ground state, giving off energy in form of fluorescence or vibrational energy (photophysical reaction) or undergoes intersystem crossing to the longer lived (10^−3^ seconds) triplet excited state (photochemical reaction). The interaction of the triplet sensitizer with surrounding molecules results in two types, I and II, of competitive photochemical reactions mediating PDT cytotoxic effects [100]. In type I reaction, the activated PS directly interacts with a substrate, such as membrane lipids or other molecules, which then may react with oxygen generating free radicals (OH^–^), peroxides (H_2_O_2_) and superoxides (O_2_^−^). On the other hand, type II photochemistry involves direct energy transfer from the excited PS to ground-state molecular oxygen, to mainly form excited-state singlet oxygen (^1^O_2_), the most important ROS mediating PDT cytotoxicity through the rapid and indiscriminate oxidative reaction with biological molecules like lipids, proteins or nucleic acids (Figure 2). The lifetime (≤50 ns) and the diffusion length (≤20 nm) of ^1^O_2_ in cellular compartments are very limited and influence the site of damage that, in turn, strictly corresponds to the PS localization [101]. 

## 7. Autophagy in PDT-Photosensitized Cells

Reportedly, autophagy has been suggested as a common outcome concomitant and/or precedent and/or consequent to apoptosis in PDT, occurring in a variety of cell lines photosensitized with a broad spectrum of PSs. The kinetic of apoptosis/autophagy switch strictly depends on cell type, photosensitizer type and concentration and light dose. Autophagy in PDT plays a prosurvival role in apoptosis competent cells and a prodeath role in deficient ones (reviewed in Reiners *et al.* [102]). Mitochondrial-, ER- and lysosomes-localized PSs are involved in PDT-induced autophagy [103,104,105].

In particular, ER and/or mitochondria photodamage triggers a prosurvival autophagic response to recycle injured organelles [105,106]; conversely, PSs localizing and damaging lysosomes block autophagosome processing leading to autophagy inhibition.

Noteworthy, in Dini’s laboratory it is demonstrated that Rose Bengale Acetate, a PS localizing in ER only after redistribution from its perinuclear primary damage site induces autophagic cell death [107,108] in addition to apoptosis [109,110] in human epitheloid cervix carcinoma HeLa cells. 

Highly cytotoxic ^1^O_2_ is the main oxidative agent mediating PDT cellular damage. However, among ROS generated during photodynamic protocol, hydrogen peroxide (H_2_O_2_), formed downstream of ^1^O_2_, can induce autophagy in its cytoprotection/cytotoxicity “double face” role [111]. In this context, autophagy-lysosomal system represents a second line of defense against ROS in mammalian cells [112] in addition to cellular detoxifying and antioxidants enzymes and agents [113].

Then, all together ROS type, oxidative injury and molecular targets involved affect the role of autophagy in cancer therapies, such as PDT [112]. Particularly, mounting evidences suggest that PDT stimulates autophagy occurrence by photooxidation/inactivation of autophagic negative regulators, such as Bcl-2 and mTOR proteins [114,115,116,117] rather than key autophagic proteins, such as Beclin1, Atg5 and Atg7. 

The Bcl-2 family proteins are well-characterized key regulators of apoptotic cell death, conversely their effect on non-apoptotic cell death is less clear. However, it seems probable that the balance between pro- and anti-apoptotic Bcl-2 members act as rheostat also in autophagic cell death [68,82].

Many ER and/or mitochondrial localized PSs commonly used in PDT studies (*i.e.*, 9-capronyloxytetrakis (metoxyethyl) porhycene, CPO, tin ethyl etiopurpurin, SnET2, benzoporphyrin derivative, BPD, and silicon phtalocyanine, Pc4) photodamage Bcl-2 and/or Bcl-x_L_ proteins in a light-dose and cell-type dependent manner, leading to the loss of both their anti-apoptotic [114,115,116] and anti-autophagic functions, mediated by binding to the pro-autophagic Beclin1 protein [82]. In fact, in mouse leukemia L1210 cells, photosensitization with CPO stimulates autophagy induction likely by disrupting the association Bcl-2/Beclin1 [106]. On the other hand, in human breast cancer MCF-7v cells lacking in caspase-3, and in human prostate cancer DU145 cells lacking in Bax, the Bcl-2 overexpression does not protect against autophagy after Pc4-PDT [118]. Conversely, in MCF-7c3 stably overexpressing human pro-caspase-3 and Chinese hamster ovary CHO 5A100, the overexpression of Bcl-2 provides autophagic protection occurrence [119,120]. 

In this context, it should be considered the cell apoptosis competence or deficiency. In fact, DU145 and MCF-7v cells die after Pc-PDT suggesting not only the pro-death role of autophagy, but also its apoptosis occurrence independence [115]. Moreover, Bax results ineffective in autophagic cell death [121] as also demonstrated in murine embryonic fibroblasts Bax^−/−^Bak^−/−^ double-knockout (Bax^−/−^Bak^−/−^ DKO MEFs). 

Particularly, dose dependent autophagy occurrence after photosensitization was early observed in both procaspase-3-deficient MCF-7v and procaspase-3-expressing MCF-7c3 cells, implying that procaspase-3 is not required for this process. Both apoptosis and autophagy occur in MCF-7c3 cells; however, the apoptotic cell death is the predominant mechanism [122]. Moreover, MCF-7c3 cells are more sensitive to PDT damage than MCF-7v cells indicating that the apoptotic pathway is faster than non-apoptotic one [123]. 

Similarly, in ER-localized hypericin-sensitized Bax^-/-^Bak^-/- ^DKO MEFs, TEM analysis reveals the hallmarks of early-stage macroautophagy, absent in wild-type MEFs (WT MEFs). Moreover, the inhibition of autophagy with Wortmannin prevents cell death, while the inhibition of caspase-dependent apoptosis with z-VAD does not prevent autophagy suggesting that PDT-induced autophagy in WT MEFs is a separate route of cell demise and the independence of the two signaling pathways [104]. In keeping with this result, also in RBAc-photosensitized HeLa cells autophagy plays a pro-death role independently from apoptosis [108].

The “double face” role of autophagy has been demonstrated in 5-aminolevulinic-based photodynamic therapy (5-ALA-PDT) [124,125].

Autophagy is the predominant form of cell death in rat adrenal medulla pheochromocytoma PC12 and in human lung adenocarcinoma CL1-0 cells after 5-ALA-PDT. Indeed, in these cell lines, the photokilling is prevented by inhibiting autophagy and not apoptosis. Moreover, autophagy induction requires the activation of AMP-activated protein kinase (AMPK) signaling cascade, but not of mitogen-activated protein kinase (MAPK) one [124]. AMPK plays a critical role in response to cellular stress [126] and it is implicated both in anti- [127,128] and pro-apoptotic effects [129,130] and in autophagy [131,132], implying that it could be a potential target in cancer treatment.

Conversely, 5-ALA-PDT stimulates cytoprotective autophagy against PDT-mediated necrosis in human glioblastoma LN18 and U87 cell lines by activation of NF-_k_B (nuclear factor kappa-light-chain-enhancer of activated B cells), nuclear factor implicated in many cellular processes such as immunity, apoptosis, angiogenesis and proliferation [125].

Among negative regulators of autophagy, the rapid shutdown of the Akt-mTOR pathway promotes the development of autophagy [133].

Recently, Weyergang *et al.* [117] demonstrated that, in colon adenocarcinoma WiDr cells, the amphiphilic endolysosome-localizing PS disulfonated aluminum phthalocyanine (AlPcS_2_) targets photodamage in mTOR signaling network. Particularly, AlPcS2-PDT downregulates the levels of Ser(2448) phosphorylated mTOR (p-mTOR), total mTOR and phosphorylation of ribosomal S6 (p-S6) immediately after light exposure in a dose-dependent manner. 

Since autophagy induction is a common outcome in PDT, the key autophagosomes assemblying proteins are unaffected in PDT protocols performed with ER and mitochondrial localized PSs.

PDT performed with the mitochondria localized PS benzoporphyrin derivative monoacid ring A (BPD) does not photodamage Beclin1, Atg5 and Atg7 proteins in rat leukemia L1210 cells. Likewise, neither Beclin1 nor Atg5 were photodamaged in HeLa cells and MEFs photosensitized with hypericin (Hyp) [102]. 

ER-localized PSs-PDT, such as Hyp, profoundly perturbs ER-Ca^2+^ homeostasis by photodamage of sarco(endo)plasmic-reticulum Ca^2+^-ATPase2 (SERCA2) pump triggered immediately after irradiation by ^1^O_2_ in HeLa cells, human bladder carcinoma T24 cells and MEFs. SERCA2 photodamage and ER-Ca^2+^ depletion commit apoptotic-defective cells to autophagy, suggesting its pro-death role [104]. Recently, another ER-associated protein, the Inositol trisphosphate Receptor (IP3R) involved in cytoplasmic material sequestration during cell death [41], is affected by PDT in a cell line- and PS type-dependent manner. In fact, photodynamic treatment elicited by Hyp does not photodamage IP3R in MEFs, contrary to Pc4-PDT in MCF-7 cells [102,104]. 

The relationship between apoptosis and autophagy and the consequent switch are cell line- and *stimulus*-dependent. In this context, Atg5 and Atg7 proteins play a key role: by silencing Atg5 and Atg7 genes, autophagy is curtailed, promoting photokilling. Kessel and coworkers [105] elicit CPO-photosensitization in L1210 cells, with and without stable knockdown of Atg7. The response of cells is different in relation to *stimulus* intensity. Particularly, after low PDT dose, the autophagy-competent L1210 cells are more resistant to death than autophagy-deficient ones; conversely, cytotoxicity is similar after high PDT dose. In this paradigm, Atg5 gene silencing or 3-MA use attenuate macroautophagy triggered along with apoptosis by ER-localized PSs and increase the cytotoxic effects of PDT. A similar result is achieved up-regulating the CMA in unstressed Atg(−/−) cells, disclosing that CMA, whose role in ROS-based cancer therapies is largely unexplored, could be the dominant defense mechanism against PDT [134].

The cytoprotective role of autophagy and its dependence on PDT dose were also reported in 1c1c7 murine hepatoma cells photosensitized by BPD (Verteporfin). Varying BPD dose, Andrzejak *et al.* [135] demonstrated the protective effect of autophagy in WT 1c1c7 cells. Moreover, the extent of cell death is greater in Atg7 knockdown 1c1c7 (KD) cells both in low and high BPD dose. WT cells present a high number of autophagosomes than KD ones showing apoptotic morphological features. Very high BPD dose (until 10 µM) inhibits autophagosomes formation both in WT and KD cells, as demonstrated also in MCF-7 cells [136]. 

A change in cell death profile, apoptosis/autophagy and *vice versa*, following photodynamic treatment has been also demonstrated after 5-ALA photosensitizition of the U2OS osteosarcoma cell line deficient in receptor-interacting protein 3 (RIP3), a key molecule in necroptosis [137,138,139] and apoptosis [140,141]. The expression of RIP3 in U2OS (RIP3-U2OS) exhibits a higher apoptosis activation after 5-ALA-PDT, but also a better survival than wild type U2OS. This result is consistent with the enhancement, in RIP3-U2OS, of autophagic flux suggesting a cytoprotective role of autophagy in this cell line [142]. However, to date no link between RIP3 and autophagy induction/regulation has been demonstrated. 

Independently from their localization, PSs localized in ER, mitochondria or endosomes/lysosomes, are all capable to induce autophagy and the consequent autophagosomes formation. However, the subsequent processing of autophagosomes can be different. In fact, the completion of autophagic process requires autophagolysosomes formation following the autophagosome-lysosome fusion that can be direct or mediated by the previous amphisome formation. PSs taken up into the cell by endocytosis overall localize in the structures above mentioned, targeting the photosensitization upon irradiation [143]. Endosomally/lysosomally PSs, e.g., mono-L-aspartyl chlorin e6 (Npe6) [144], Meso-tetra-(p-sulphophenyl) porphine (TPPS_1–4_) [116], AlPcS_2–4_ [145], can localize to the target organelles membrane or concentrate in the organelle’s matrix, inducing membrane damage or lysosomal enzymes photoinactivation respectively. The lysosomal membrane breakdown affects the autophagic flux; the release of proteolytic enzimes triggers mitochondrial apoptosis by Bid cleavage [144]. In fact, in murine hepatoma 1c1c7 cells illuminated after Npe6 loading, the autophagosomes formation is early observed in parallel with pro-caspase-3 and -9 activation [146]. These data suggest both the autophagy induction and the failure of autophagic process completion. Alternatively, the destruction of lysosomal proteases negatively affects degradation of the cargo in autolysosomes [102].

*In vivo*, PSs accumulate in tumor cells and/or in neoplastic vasculature leading to direct cell killing and vascular shutdown respectively [147]. The latter induces autophagy consequent to nutrient depletion and hypoxia [148,149]. The hallmarks of autophagic process, such as the appearance of vacuoles and lipofuscin bodies in the cytoplasm, were observed in rat orthotopic superficial bladder cancer phototreated with hexyl 5-aminolevulinate (HAL) [150]. The *in vivo* pro-death or pro-survival autophagy role remains to be elucidated.

**Figure 3:**
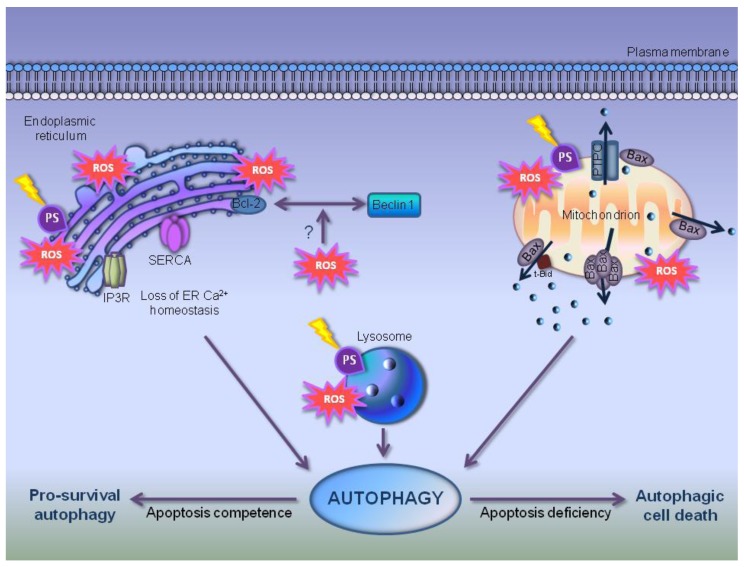
Autophagic-induced photodamage triggering cytotoxicity/cytoprotection in cancerous cells. In PDT, ROS generated by different PSs localizing in mitochondria, Endoplasmic Reticulum (ER) and lysosomes trigger autophagy as part of a death or survival pathway in relation to neoplastic cells apoptosis deficiency or competence respectively. Probably, photosensitization damages and/or inactivates anti-apoptotic Bcl-2 and Bcl-x_L_ proteins and/or causes their dissociation from pro-autophagic Beclin1 protein, favoring the induction of autophagy. Moreover, PDT photodestroys proteins (Bax oligomers, Bax-associated PTPC (Permeability Transition Pore Complex), Bax monomer and Bax associated tBid, truncated Bid) forming pores on mitochondrial membranes, altering the correct solutes flux between mitochondria and cytoplasm, SERCA pump and IP3Rs in ER, inducing the loss of ER Ca^2+^ homeostasis. Finally, PSs localizing in lysosomes breakdown their membrane or inactivate the matrix proteases.

A schematic depiction of autophagic induction by photodynamic damage is reported in Figure 3.

## 8. Does Autophagy Concur to Elicit an Immunogenic Impact in Photosensitized Cells?

One of the main dangerous hallmarks of cancer cells is the resistance to cell death. In fact, during their evolution to the malignant state, tumor cells evolve multiple ploys to ensure the survival [151], by acquiring the ability to replicate also in an inflamed microenvironment, to evade immune recognition and to suppress immune reactivity [152].

Therefore, an attractive strategy that holds the highest therapeutic value in the clinical management of the neoplasia should combine the restoration of cell death, by increasing the susceptibility of cancer cells towards death, and the retrieval of the immune surveillance, enhancing the immunological recognition of tumor cells [153].

This may be achieved by favoring cancer therapies that induce Immunogenic Cell Death (ICD) and avoiding cancer regimens mediating immunosuppressive side effects, such as myelosuppression or thymolysis.

It is still enigmatic under which circumstances cellular demise induces an immune response against dying tumor cells or rather remains immunologically silent.

Among cell death modalities, apoptosis has been unanimously considered as an immunologically silent cell death [154] until the recent invalidation of this concept [66,155]; conversely necrosis is still retained as an immunologically harmful PCD [156]. The immunogenic impact of autophagy is still a matter of debate, due to its conundrum survival/death onset [157]. Nevertheless, even ACD has been shown to be capable of stimulating some immunological flutters [158].

The immunogenicity of the cells dying *via* apoptosis, necrosis or autophagy is mediated by a series of subtle *spatio*/temporally changes in the composition of the cell surface and secretion of soluble molecules allowing the immune effectors, primarily dendritic cells (DCs), to ‘sense’ immunogenicity [155].

Intracellular molecules, categorized as Damage-Associated Molecular Patterns (DAMPs) or alarmins, normally hidden within live cells, are released from or exposed at the surface of dying cell to lead ICD in terms of apoptotic bodies engulfment, DC activation and maturation, antigen processing and T cell activation [159].

DAMPs exposed on plasma membrane (e.g., calreticulin (CRT), HSP70 and HSP90), or extracellularly secreted (e.g., HMGB1, uric acid, IL-1α and other pro-inflammatory cytokines) exert an immunostimolatory/immunomodulatory effect by interacting with pattern-recognition receptors (PRRs) expressed on innate immune cells [160]. The actual diversity of DAMPs, also including end-stage degradation products (e.g., ATP, DNA and RNA) and extracellular matrix compounds (e.g., hyaluronan, heparan sulfate and degraded matrix constituents), may depend on factors, such as the type of cell death, cell-type and tissue injury [161].

Particularly, it has been demonstrated that autophagy accompanying cell death induces the secretion of HMGB1 [158,162], whose cytosolic form regulates starvation-induced autophagy [163]. The HMGB1 form released by autophagic cells promotes cancer cell survival by limiting apoptosis in HCT116 human colon cancer cells, revealing the *alter ego* aspect of HMGB1 in immunogenic impact of cancer cells death [158]. 

The appealing idea of immunogenic cancer cell death demands screening and selection of newer chemotherapeutic agents/modalities capable of sustaining a particular *spectrum* of DAMPs. 

PDT has considerable beneficial immunomodulatory potential in terms of cancer disease management, since it is able in inducing expression, exposure/release of certain DAMPs [160] and activation of immune system [164]. 

As reported in several papers, PDT is a therapeutic cancer approach inducing cell death accompanied by or elicited by autophagy [94,102,112,134]. Reportedly, Garg *et al.* [165,166] and Korbelik *et al.* [167] suggest that PDT, based on hypericin (Hyp) and Photofrin phtosensitization respectively, can be considered an ICD inducer as anthracyclines, oxaliplatin and γ-irradiation. Specifically, Hyp-PDT potently stimulates CRT exposure and ATP release in T24 cells and murine colon carcinoma CT26 cells, subsequently phagocytosed by macrophages and DCs with high efficiency. Moreover, of note is that ATP release in Hyp-PDT depends on the PERK activity able, in turn, to stimulate autophagy [166]. This result suggests that autophagy could be involved in Hyp-elicited ATP secretion and could stimulate ICD. 

In parallel, high-inflammatory PDT regimens induce acute inflammation characterized by increased expression of pro-inflammatory cytokines including TNF-α, IL-1β and IL-6 [168], adhesion molecules E-selectin and ICAM-1, and the rapid accumulation of leukocytes into the treated tumor area [169].

PDT enhancement of anti-tumor immunity appears to involve the stimulation of DCs by dying tumor cells [170]. Indeed, the incubation of PDT-treated tumor cells with immature DCs implies an enhanced DC maturation, activation and ability to stimulate T cell activation [171].

## 9. Conclusions

The data reported here stress the relevance of autophagy in PDT-induced cell death, although its exact role in the photodynamic process still remains unclear.

PDT is a highly efficient cancer modality, mediating death of neoplastic cells. It can elicit all three forms of PCD in relation to the site of the initial photodamage. PSs localizing mitochondria, and/or ER and lysosomes are very efficient in inducing autophagy, whose role and interplay with apoptosis primarily depends on the PDT dose. In fact, at low dose, autophagy ensures the removal of damaged cell components maintaining cell viability; whereas conversely at high PS dose, apoptosis overwhelms autophagy and triggers cell death. 

Interestingly, in apoptosis-defective cells, autophagy is crucial in determining cell sensitivity to PDT. This offers hope that, during photosensitization, it could be modulated in relation to apoptotic machinery bypassing the resistance of cancerous cells and, finally, adding value to PDT therapeutic potential.
